# 2489. An Innovative Capacity-Building Approach to Enhance Acute Flaccid Paralysis Surveillance During a Wild Poliovirus Type 1 Outbreak in Malawi, October 2022 - March 2023

**DOI:** 10.1093/ofid/ofad500.2107

**Published:** 2023-11-27

**Authors:** Edward Dagoe, Austin C Zgambo, Farrell A Tobolowsky, Daniel D Mapemba, Alinune Nathanael Kabaghe, Terence Tafatatha, Mtisunge Yelewa, Mike N Chisema, Grace M Funsani, Brenda Mhone

**Affiliations:** Centers for Disease Control and Prevention, Atlanta, Georgia; Public Health Institute of Malawi, Lilongwe, Lilongwe, Malawi; CDC, Atlanta, Georgia; Ministry of Health, Public Health Institute of Malawi, Lilongwe, Lilongwe, Malawi; US CDC, Malawi, Lilongwe, Lilongwe, Malawi; U.S. Centers for Disease Control and Prevention (CDC) Malawi, Lilongwe, Lilongwe, Malawi; Ministry of Health, Public Health Institute of Malawi, Lilongwe, Lilongwe, Malawi; Ministry of Health, Lilongwe, Lilongwe, Malawi; Ministry of Health, Public Health Institute of Malawi, Lilongwe, Lilongwe, Malawi; Ministry of Health, Lilongwe, Lilongwe, Malawi

## Abstract

**Background:**

After 30 years without a poliovirus case, a wild poliovirus type 1 outbreak was declared in Malawi on 16^th^ February 2022. To improve detection of poliovirus transmission, the country implemented enhanced AFP surveillance by deploying Frontline Field Epidemiology Training Program (FETP) graduates who had received a three-month general epidemiology training. We describe the results from October 2022 to March 2023.

**Methods:**

Ten districts with increased risk of polio transmission and suboptimal AFP surveillance performance indicators were identified for enhanced AFP surveillance. One FETP graduate already working in each priority district was selected to be trained on poliovirus epidemiology and AFP surveillance. FETP graduates conducted active case finding for AFP cases in 100 priority health facilities (defined by epidemiologic risk and population size), provided on-the-job training for health care workers, and sensitized community volunteers and traditional healers on AFP recognition and reporting. A senior technical coordinator provided supportive supervision to FETP graduates in all 10 districts.

**Results:**

During the observation period, FETP graduates completed 811 (44%) of 1839 visits to priority health facilities and reported 84% (59/83) of all AFP cases in the priority districts. Eleven additional AFP cases were retrospectively identified during FETP graduates’ health facility register reviews. FETP graduates trained 5,047 health care workers and educated 2,093 community volunteers.

**Figure:**
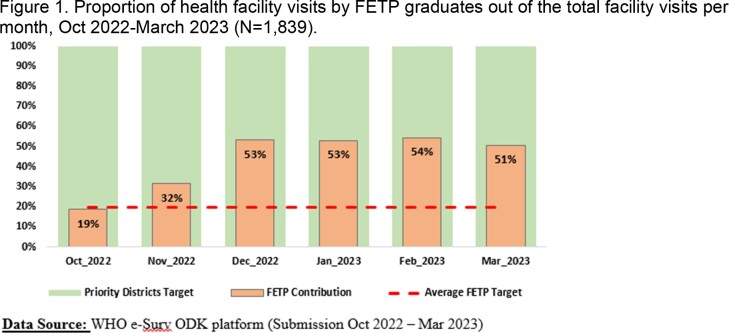

**Figure 2.**
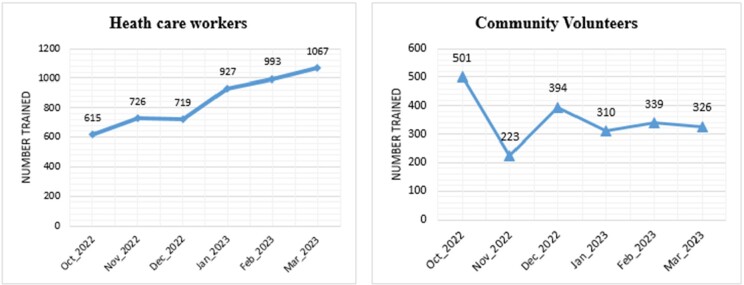
Number of health care workers and community members that were educated by FETP graduates on AFP case detection and notification in the priority districts by visiting month, October 2022 - March 2023.

**Conclusion:**

The enhanced surveillance contributed to almost half of all active case search visits, detected most AFP cases in the identified priority districts, and educated the community to improve poliovirus identification and reporting. Including FETP graduates to train the local public health workforce is an innovative approach in Malawi to build capacity, rapidly mobilize in-country staff, optimize the available resources, and strengthen surveillance during a novel disease outbreak.

**Disclosures:**

**All Authors**: No reported disclosures

